# Limits on phenological response to high temperature in the Arctic

**DOI:** 10.1038/s41598-022-26955-9

**Published:** 2023-01-05

**Authors:** Sarah C. Elmendorf, Robert D. Hollister

**Affiliations:** 1 Institute of Arctic and Alpine Research, University of Colorado, Boulder, CO 80309-0450 USA; 2 Department of Ecology and Evolutionary Biology, University of Colorado, Boulder, CO 80309-0450 USA; 3 Biology Department, Grand Valley State University, 1 Campus Drive, Allendale, MI 49401 USA

**Keywords:** Biodiversity, Climate-change ecology

## Abstract

Tundra plants are widely considered to be constrained by cool growing conditions and short growing seasons. Furthermore, phenological development is generally predicted by daily heat sums calculated as growing degree days. Analyzing over a decade of seasonal flower counts of 23 plant species distributed across four plant communities, together with hourly canopy-temperature records, we show that the timing of flowering of many tundra plants are best predicted by a modified growing degree day model with a maximum temperature threshold. Threshold maximums are commonly employed in agriculture, but until recently have not been considered for natural ecosystems and to our knowledge have not been used for tundra plants. Estimated maximum temperature thresholds were found to be within the range of daily temperatures commonly experienced for many species, particularly for plants at the colder, high Arctic study site. These findings provide an explanation for why passive experimental warming—where moderate changes in mean daily temperatures are accompanied by larger changes in daily maximum temperatures—generally shifts plant phenology less than ambient warming. Our results also suggest that many plants adapted to extreme cold environments may have limits to their thermal responsiveness.

## Introduction

The tundra region is warming at more than twice the world average, with concomitant increases in plant productivity, shifts in vegetation structure, and earlier onset of spring phenological events evident in recent years^1–4^. Access to remote research locations hampers field research in tundra regions, which are underrepresented in climate change and phenological research, relative to their geographic size^5^. As a result, the environmental cues that trigger phenological change in tundra plants are less well understood than the combination of chilling, forcing and photoperiod effects that cue spring phenology in temperate regions^6^. Tundra plant phenological development is typically delayed by late snowmelt but the relationship between snowmelt timing and phenological events may vary greatly between years. For example, phenological transitions often occur more rapidly after snowmelt in late snowmelt years or locations^7–9^, presumably because the relatively warmer air temperatures experienced after late snowmelt speed development.

The combined effects of snowmelt and temperature can be described by a growing degree day (hereafter GDD) index (Eq. 1), which typically explains the timing of tundra phenological events better than the date of snowmelt or temperature alone^10–13^.

1 $$GDD=\left\{\begin{array}{ll}0&\quad {\text{if }}T\le 0{\text{ or snow-covered}}\\ T&\quad {\text{if }}T\ge 0{\text{ and snow-free}}\end{array}\right.$$

where T is mean daily (or hourly) temperature. However, problems have been noted with the GDD model for tundra plant phenology. If GDD consistently explained phenological dates, we would expect the heat sums accumulated or thermal time at key phenological events to be constant, yet considerable variability in the GDD corresponding to key phenological transitions has been observed over time and with warming and snow manipulation experiments^13–15^.

The GDD model assumes that there is a lower limiting temperature for tundra plant development but that increasing temperature above the baseline consistently advances phenological development. In tundra environments growth typically begins around 0 °C^16^ and the ambient temperatures are historically considered far lower than the optimum temperature for photosynthesis in these cold adapted regions^17,18^. More recent studies, however, indicate that at high latitudes optimum temperatures for photosynthesis may be as low as 10 °C^19^, and that high Arctic plants exhibit stress at high temperatures^20^. These recent observations of lower than anticipated optimums coupled with regional warming suggest that phenological modeling may need to account for high temperatures similar to agricultural systems where high and low temperature thresholds are used to predict growth^21,22^. To our knowledge, the hypothesis that there is a maximum limiting temperature for tundra plant phenology has not been explored. In contrast, model systems in lower latitudes have found either saturating phenological response to increasing temperature^23^ or in some cases, delayed phenology with very high temperatures^24,25^.

Decreased phenological sensitivity to hot versus warm temperatures could explain observed discrepancies in the effects of ambient versus experimental warming on plant phenology^14,26^. Under passive experimental warming conditions, tundra plants tend to exhibit advanced phenology in terms of calendar time (i.e. flower at an earlier day of year) but delayed flowering in terms of thermal time (i.e. have greater number of accumulated growing degree days at flowering under experimental warming)^14^. Because experimental warming generally results in a larger daily range of temperatures, with higher daily maximums than what typically occurs under ambient conditions, a large fraction of the heating that occurs in warming chambers may exceed maximum limiting temperatures for tundra plants. If this is the case, species with phenological sensitivities that saturate in response to high temperatures would be expected to accumulate more thermal time prior to flowering in warming chambers than under ambient conditions. Similarly, saturating impacts of high temperatures would cause experimentally warmed plants to show less phenological sensitivity than plants exposed to ambient temperature change when sensitivity is calculated as responsiveness to mean daily temperatures, since warming chambers increase the maximum daily temperature more than the mean^27^.

We used more than a decade of seasonal flower counts of 23 plant species distributed across four plant communities, together with hourly canopy-temperature records, to understand the phenological response of tundra plants to temperature. Specifically, we ask: Does increasing warmth always advance flowering phenology or are there temperatures above which species lack the capacity to further advance? Do these relationships differ between plants adapted to higher Arctic and lower Arctic regions?

To address these questions, we used generalized additive mixed models (GAMMs) to fit the seasonal trajectory of flowering to accumulated growing degree sums under ambient conditions, varying the maximum hourly temperature that contributes to growing degree day sums from 0 to 20 °C (GDD_max_, Eq. 2).

2 $${GDD}_{max}=\left\{\begin{array}{ll}0&\quad {\text{if }}T\le 0{\text{ or snow-covered}}\\ T&\quad {\text{if }}T\ge 0{\text{ and }}T\le {T}_{max} {\text{ and snow-free}}\\ {T}_{max}&\quad {\text{if T}}\ge {T}_{max} {\text{ and snow-free}}\end{array}\right.$$

where T is mean hourly temperature and T_max_ is the maximum temperature threshold above which additional warming does not contribute proportionally to phenological development. The temporal (hourly) and spatial (temperature measured at the height of the plant canopy) resolution of temperature records is necessary to perform these calculations particularly in systems where freezing temperatures are common^28,29^. Diurnal fluctuations in daily temperature can lead to substantial discrepancies in thermal time when calculated based on hourly versus daily temperatures^30^. Similar issues can arise when temperatures are measured at the typical height of meteorological stations (2 m) rather than where the plants exist (0–20 cm).

To estimate the maximum temperature threshold, we compared models of GDD_max_ calculated with maximum thresholds from 0 to 20 °C; the model with the lowest Akaike information criterion (AIC) values was considered the best fit. We then compared the ability of the resulting models, fitted using plants observed under ambient conditions, to predict the timing of flower development in plants that had been experimentally warmed. Specifically, we compared models using day of year (DOY), GDD and GDD_max_.

## Results

**Figure 1:**
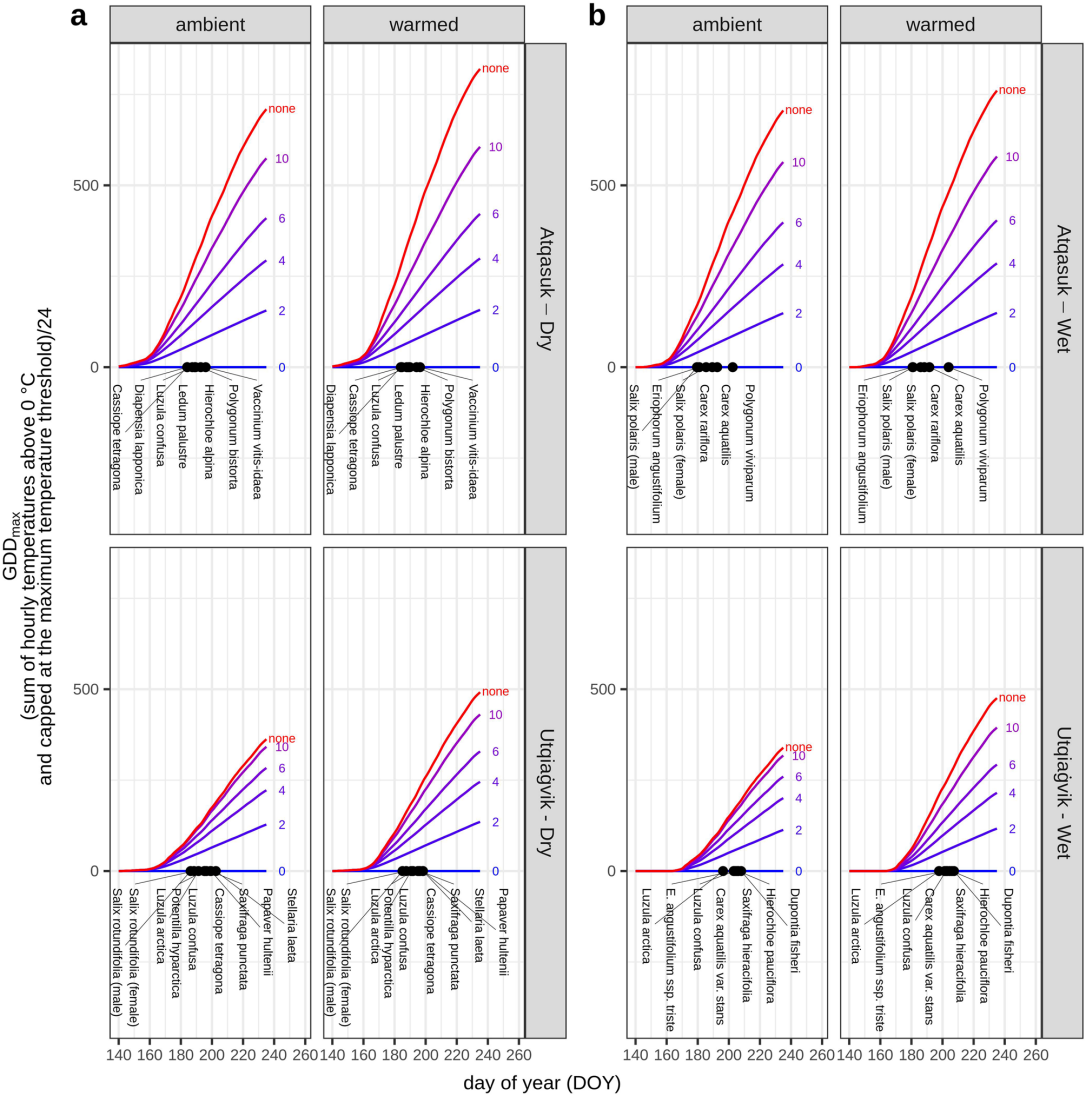
Growing degree day (GDD) sums calculated using different maximum temperature thresholds. Lines represent GDD_max_ on a given day (averaged across years) for each location in both ambient and warmed plots, calculated with a maximum threshold of 0, 2, 4, 6, 10 °C or no threshold. The spread between lines is smaller at higher than lower thresholds because of the relatively low frequency of higher temperatures. For comparison with plant phenology, the rugplot shows the mean day of flowering averaged across years for plants monitored at each location.

**Figure 2:**
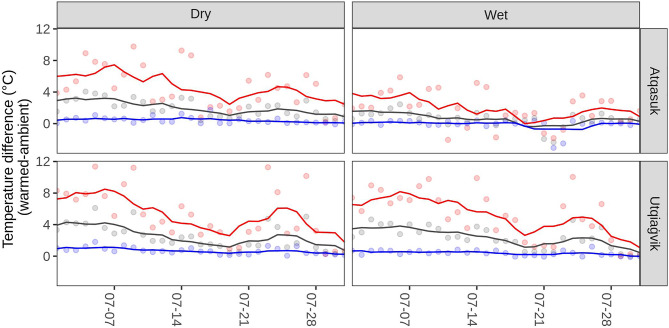
Daily variability of passive experimental warming. Points show the daily difference in minimum (blue), maximum (red) and mean (grey) temperatures in July of 2008 at each location; lines show the 7-day rolling average. Passive warming with open topped chambers varies based on weather conditions but is generally greatest when solar intensity is highest resulting in increased daily maximums.

The higher Arctic region (Utqiaġvik) was consistently colder than the lower Arctic region (Atqasuk). The accumulated GDD_max_ calculated using different maximum thresholds show minor differences when the threshold was above 10 °C, especially at Utqiaġvik, because temperatures above 10 °C occur infrequently (Fig. 1). The effectiveness of the experimental warming treatments varied seasonally, with larger effects earlier in the season when solar intensity is highest. Notably, warming treatments increased daily maximum temperatures more than mean temperatures and did not substantially alter daily minimum temperatures (Fig. 2). As a result, experimental warming increased accumulated GDD_max_, but the differences between treatment and control plots was more pronounced when higher GDD_max_ threshold was considered (Fig. 1).

**Figure 3:**
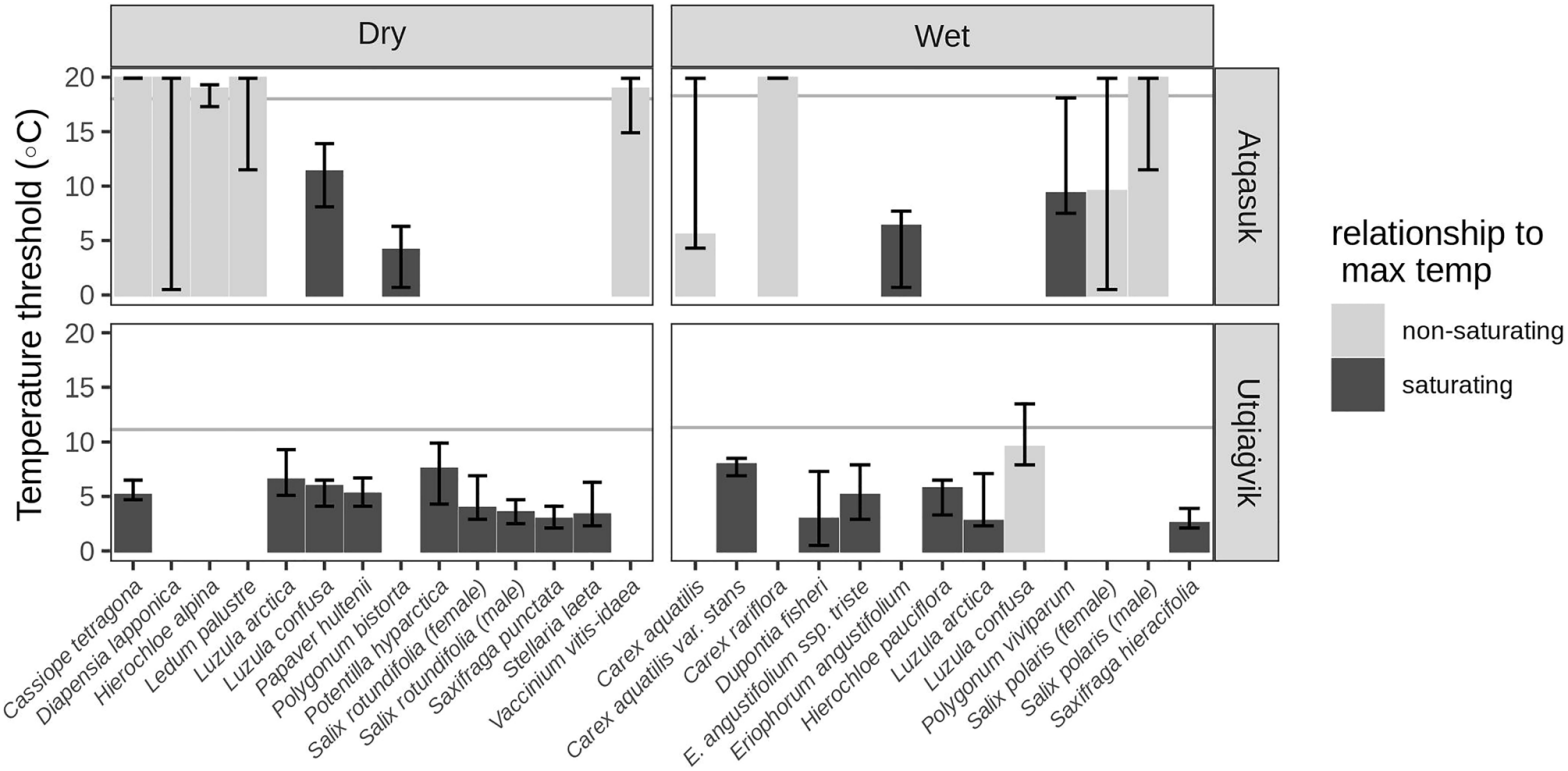
Estimated maximum temperature thresholds of tundra plants. Thresholds were estimated as the lowest AIC values when comparing generalized additive mixed models of GDD_max_ (calculated from 0 to 20 °C) with flower counts of the plants growing in ambient conditions observed at each location. The height of bars indicates the median estimated threshold after refitting the models omitting a single year in each iteration; error bars show range (min and max). A dark fill color indicates that the species showed a saturating phenological response to increasing temperature (where accounting for higher temperatures decreases the quality of the model) and show evidence for a maximum threshold within the range of temperatures commonly observed (see Supplemental Fig. 2 for individual fits). A light fill color indicates a lack of consistent evidence for a maximum threshold within the range of temperatures commonly observed. Below the horizontal line represents 95% of the observed hourly summer air temperatures at the location.

Plants at the higher Arctic locations (Utqiaġvik) consistently showed saturating phenological responses to high temperature (Fig. 3). Estimated maximum temperature thresholds around 5 °C indicate that hot days at this location advance phenology no more than a warm day. At the lower Arctic locations (Atqasuk), saturating phenological responses to maximum temperatures were less common and when they occurred the thresholds were generally above 10 °C. There were no clear differences between wet and dry locations (Fig. 3).

**Figure 4:**
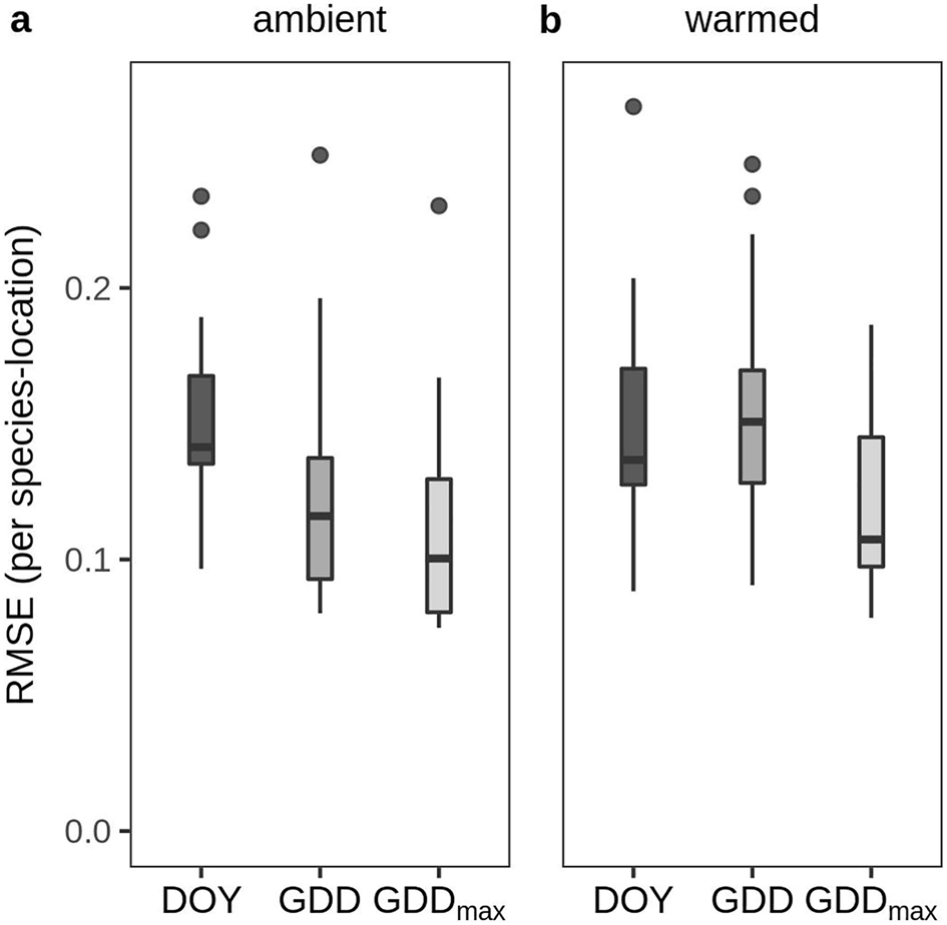
The predictability of flowering. Box-plots compare the utility of models using day of year (DOY), accumulated growing degree days (GDD) or accumulated growing degree days with an estimated maximum temperature threshold GDD_max_ to predict flowering in ambient (**a**) and warmed (**b**) plots. Values represent the root mean squared error (RMSE) calculated for each species at each location based on the difference between the predicted and actual number of flowers in each survey, normalized by the total number of flowers counted in each year. Lower RMSE implies a better model. The maximum temperature thresholds used in the GDD_max_ models were estimated using only observations from plants in ambient conditions; only those species that showed a saturating relationship to temperature are included here (male and female *Salix* and subspecies were treated as separate species).

Models that used a calibrated GDD_max_ value to predict the seasonal timing of flowering performed better than models using a traditional GDD summation (that do not incorporate a maximum value) and better than models using day of year (DOY) alone (Fig. 4a). The difference in predictive performance between GDD and GDD_max_ models was more pronounced when examining predictions of the timing of flowering for plants that had been experimentally warmed (Fig. 4b) than comparing among years in control plots only. These results are congruent with the amplified differences in accumulated GDDs between experimentally warmed and control plots at high GDD_max_ thresholds (Fig. 1).

## Discussion

Evidence that plant phenology will keep pace with a rapidly warming climate warming is mixed. A study in boreal peatlands found linear shifts in phenological development with increases in temperatures of up to 9 °C^31^. In contrast, a growing collection of studies of temperate trees suggest that plant phenological responses to climate warming may be nonlinear, either due to co-limitations of chilling and daylength^32^ or decreasing effects of increasingly high temperatures^33^. Here we show that phenological development of many tundra plant species growing in cold regions does not respond linearly to high temperatures. Instead, at the high Arctic site, we found that high temperatures did not shift the seasonal reproductive cycle further than warm temperatures. These results have implications both for the interpretation of climate warming experiments and projecting the impact of near-term climate change on high latitude plant communities. Our study also provides one potential explanation for the discrepancy between temperature sensitivity of phenology as calculated from experimental warming versus long-term observations. The lack of a response to temperatures above relatively low thresholds (less than 10 °C) suggest that many tundra plants in the higher Arctic may not benefit from warmer temperatures and that they may experience heat stress similar to that of temperate plants but at much lower temperatures^34,35^.

Where biological responses to climate warming are non-linear, as seen here, the nuanced effects of climate warming are critical to forecasting change under future conditions^28^. Recent climate warming has not affected minimum temperatures and maximum temperatures equivalently. Instead, daily minimum temperatures have risen more dramatically than maximum temperatures^36^. These diurnal shifts are opposite to those typically seen under passive experimental warming, where nighttime temperatures are unaffected but daily maximums increase strongly (Fig. 2). If these global patterns also apply to near-term warming in the high Arctic, we would expect to see large phenological shifts as low-temperature conditions become increasingly infrequent. On the other hand, recent years have witnessed historically unprecedented amount of Arctic heat waves, such as those leading to explosive wildfires in Siberia and mid-summer melting on the Greenland Ice Sheet in 2021^37^. Our results suggest that reproductive phenology in the high Arctic is not similarly vulnerable to tipping point behavior and in fact may show only moderate advances during periods of extreme heat. Yet we recognize that extreme heat waves may cause harm to plant growth and seed viability^34,35,38^.

Our results exemplify why explicit consideration of fine-scale climate warming may be necessary to understanding potential climate change impacts^28^. Where biological responses to temperature increases are non-linear, heterogeneity in warming over both space (e.g. microrefugia) and diurnally (as seen here), can lead to impacts that differ from mean warming effects. Maximum temperature thresholds are commonly employed in agricultural systems to predict yield^21,39,40^; and, more recently phenology^41^. Our findings of maximum temperature thresholds for phenological development in the majority of censused taxa at a high Arctic site suggest this phenomenon should be considered more broadly in natural systems.

## Methods

### Data

Field data were collected at a higher Arctic region (Utqiaġvik, AK, USA, 71°31′N, 156°60′W) and lower Arctic region (Atqasuk, AK, USA, 70°45′N, 157°40′W). In each region we sampled two study areas (hereafter locations) situated in dry heath (dry) and wet meadow (wet) plant communities. The dry sites are drier than the surround landscape, at Utqiaġvik the dry site is on a raised beach of fine marine silts, sands, and gravels whereas at Atqasuk the dry site is on a stabilized sand dune. The wet sites are adjacent to the dry sites at approximately a meter lower elevation on the margin of a drained thaw lake and underlain by an organic layer rich in peat. Each location contains 48 permanent 1 m^2^ plots, half of which were randomly assigned to an experimental warming treatment. Warming was achieved using open-top chambers. Plots were established between 1994 and 1996.

Plant canopy temperatures in each location × treatment combination were collected using Model 107 Temperature Probe (Campbell Scientific), HOBO Temperature Logger (Onset Computer Corporation) or StowAway Temperature Logger (Onset Computer Corporation) placed in six-plate radiation shields approximately 10–15 cm above ground surface. Readings were taken every 10–60 min, averaged, and recorded hourly (Campbell Scientific CR10X Datalogger, HOBO or StowAway Temperature Logger). Where gaps existed in the hourly data, we infilled as follows: For gaps of one hour, we used the mean of the temperature during the previous and following hours. For gaps of > 1 h, we used the mean of the temperature 24 h previous and 24 h later. Snowmelt dates were assessed visually in each plot and averaged over all plots for each location and treatment for a single date per year. In some years, researchers arrived after snowmelt. In these years, snowmelt was estimated based on soil surface temperatures.

Flowers (or inflorescences, see Supplemental Table 1 for species-specific count units; referred to as flowers for simplicity) were censused ~ weekly in all plots in each location in each of 13 years (1999–2000, 2007–2008, 2010–2018). For an example of the observations included from the ambient plots for a single species at one location see Supplemental Fig. 1. On each census day, for each species in each plot, we estimated the total number of new flowers that had opened since the last survey date as the sum of non-senesced flowers plus senesced flowers minus the number of senesced flowers counted during the previous census date. We then summed these estimates over all 24 plots of each treatment at each location to generate a single measurement of new flowers per species, site, treatment, location and survey date (Eq. 3).

3 $${Flowers}_{new,t,treatment, site}= {\sum_{p=1}^{24}{Flowers}_{non{\text{-}}senesced,t,p}+({Flowers}_{senesced,t,p}-{Flowers}_{senesced,t-1,p})}$$

In some cases, the first flower census contained > 0 flowers. For these species × location × year × treatment combinations, we used a separate survey of first flowering dates per plot to estimate the last date when no plants had flowers as the 2 days prior to the first flowering date, because surveys for first flowering dates occurred roughly every other day in the early season. We included only data from species × location × year × treatment combinations where we were able to determine the true peak in flowering (the timing of flowering often varied between plots therefore if flowers only occurred in a few plots we often could not establish a reasonable pattern) and only species that met this inclusion criterion in ambient plots for at least 10 of the 13 survey years.

### Statistical analyses

While many phenological studies focus only on the first events (e.g. date of first flowering), here we model the impact of temperature on the full seasonal distribution of flowering. The first, peak and last flowering events of tundra plants are often decoupled^42^. As a result, analyzing the full distribution of events at the population level is generally recommended to better understand the demographic and ecosystem consequences of phenological shifts^43,44^.

To test the relationship between temperature and flowering, we used generalized additive mixed models (GAMMs) to fit the seasonal curves of flower counts in ambient plots to environmental forcings (GDD, GDD_max_, or DOY). Models were fit using the gamm4 package in R, using thin plate splines and a Poisson distribution. We used the log of the intercensus interval as an offset to account for irregular sampling intervals. In the absence of finer resolution on the dates during the intercensus period when each flower actually opened, we assigned the date of flowering for each census to the midpoint between census dates and calculated environmental forcings (GDD, GDD_max_ and DOY) up until the census midpoint date. For the first census (which was always 0), and for which the intercensus interval was undefined, we assigned a typical intercensus interval of 7 days. Years were treated as random effects to account for variability in flower counts among years.

To compare the models fitted to GDD_max_ with different maximum thresholds (Supplemental Fig. 2), we ran the models with thresholds from 0.5 to 20 °C (with 0.2 degree increments) and compared the resulting AIC values^45^. Lower AIC values indicate a better model with the selected maximum threshold. Species phenological sensitivities to high temperatures were characterized as either non-saturating (no consistent evidence for a maximum temperature threshold) or saturating (local minima in AIC indicating evidence for a maximum temperature threshold) based on visual assessment of the AIC profiles. All models were fit using maximum likelihood. Models were fit separately for each species × location combination (and in the case of the dioecious species of *Salix*, separately for male and female flowers).

To generate confidence intervals on the estimated maximum temperature threshold (determined by the lowest AIC values) we refit each model sequentially dropping a single year and report the resulting median, minimum and maximum (Fig. 3). We considered the estimated maximum temperature threshold meaningful if the range was within the lower 95% of the observed hourly summer air temperatures.

To compare the ability to predict flowering with DOY, GDD, and GDD_max_ we compared root mean squared errors from each model (Fig. 4). Specifically, we predicted the number of flowers that would be counted on each survey date using the fixed effects components of the fitted models and compared that to the number of flowers observed during that survey. Because the focus of our analyses was on the timing of flowering not the absolute number (the number of flowers can vary greatly among treatments and years; see Supplemental Table 1), we normalized both the predictions and measurements each year by converting each to a percentage (number of flowers counted per census day/total number of flowers counted that season for the given species in the given treatment). We estimated the maximum temperature threshold using the process described above using only observation from ambient plots. We used GDD_max_ to predict the proportion of flowers that were observed on each census day for all ambient plots and all warmed plots. We repeated the process for GDD and DOY, using only those species × location combinations that showed a saturating relationship to temperature. We summarized the overall fit of each model by averaging the calculated root mean squared error in the percentages predicted vs. observed for each species × location × year combination. A lower RMSE indicates more consistent phenological patterns in ‘climate space’ over time (ambient treatment comparison) or across treatments (warming treatment comparison), where climate space is represented by either DOY, GDD, or GDD_max_.

All analyses were conducted in R (version 3.6.3), using packages data.table (v. 1.12.8)^46^, egg (v 0.4.5)^47^, gamm4 (v 0.2-6)^48^, gridExtra (v.2.3)^49^, Metrics (v.0.1.4)^50^, NMOF (v.2.1-0)^51^, viridis (v 0.5.1)^52^ and the tidyverse suite (v1.3.0)^53^.

### Research involving plants statement

All methods were carried out in accordance with relevant guidelines and regulations. Access to the land was permitted by the North Slope Borough Planning and Community Services Department (NSB 22-213 and NSB 22-214). Plant identification was done by Robert Hollister and Christian Bay according to Hultén (1968); updated naming follows the USDA.plants.gov. Plant specimens were archived at University of Alaska in Fairbanks and Grand Valley State University Herbarium. Voucher specimens associated with the project are listed below. Further details may be found by searching the accession numbers on https://www.pnwherbaria.org and https://midwestherbaria.org.

- *Carex aquatilis* var. *stans* ALA: UAM:Herb:12943.
- *Carex aquatilis* ALA: UAM:Herb:134911.
- *Carex* *rariflora* ALA: UAM:Herb:140082.
- *Cassiope* *tetragona* ALA: UAM:Herb:60805 Vera Komarkova.
- *Cassiope* *tetragona* ALA: UAM:Herb:12945.
- *Diapensia* *lapponica* ALA: UAM:Herb:134214.
- *Dupontia* *fisheri* ALA: UAM:Herb:143579 David T Mason.
- *Eriophorum* *angustifolium* ALA: UAM:Herb:148594 Donovan Stewart Correll.
- *Eriophorum* *angustifolium* ssp. *triste* ALA: UAM:Herb:12942.
- *Eriophorum* *angustifolium* ssp. *triste* GVSC: GVSC000585 Robert Slider.
- *Hierochloe* *alpina* ALA: UAM:Herb:134414 Swanson.
- *Hierochloe* *pauciflora* WTU: 194032 Ira Wiggins.
- *Ledum* *palustre* ALA: UAM:Herb:134132.
- *Luzula* *arctica* ALA: UAM:Herb:143643 George W. Argus.
- *Luzula* *arctica* GVSC: GVSC000647 Robert Slider.
- *Luzula* *confusa* ALA: UAM:Herb:146829 K. Olson.
- *Luzula* *confusa* GVSC: GVSC000648 Robert Slider.
- *Luzula* *confusa* ALA: UAM:Herb:134395.
- *Papaver* *hultenii* ALA: UAM:Herb:20578 Ira L. Wiggins.
- *Polygonum* *bistorta* ALA: UAM:Herb:134342 Stanwyn G Shetler.
- *Polygonum* *viviparum* ALA: UAM:Herb:142964 Karl J Stone.
- *Potentilla* *hyparctica* ALA: UAM:Herb:146999 David T. Mason.
- *Salix* *polaris* UAAH: 11661 Aaron F. Wells.
- *Salix* *rotundifolia* ALA: UAM:Herb:12944.
- *Salix* *rotundifolia* GVSC: GVSC004429 Robert Slider.
- *Saxifraga* *punctata* WTU: 193224 Ira Wiggins.
- *Saxifraga* *hieracifolia* WTU: 193221 Ira Wiggins.
- *Stellaria* *laeta* ALA: UAM:Herb:143534 John G Packer.
- *Vaccinium* *vitis*-*idaea* ALA: UAM:Herb:134153.

## Supplementary Information

Supplementary Information.

## Data Availability

The data and analysis code used in this study are available at https://doi.org/10.5281/zenodo.7474054.
