# Supplementary material for: Limits on phenological response to high temperature in the Arctic
Source: Sci Rep. 2023 Jan 5;13:208. doi: 10.1038/s41598-022-26955-9 (PMC9814414; doi:10.1038/s41598-022-26955-9)

**SUPPLEMENTAL MATERIAL:**

**Supplemental Table S1.** The maximum number of flowers or inflorescences counted across all the control plots during a single census in a given year for each species by location.

| **Species by location** | **M** | **1999** | **2000** | **2007** | **2008** | **2010** | **2011** | **2012** | **2013** | **2014** | **2015** | **2016** | **2017** | **2018** |
| --- | --- | --- | --- | --- | --- | --- | --- | --- | --- | --- | --- | --- | --- | --- |
| **Atqasuk - Dry** |  |  |  |  |  |  |  |  |  |  |  |  |  |  |
| *Cassiope tetragona* | F | 1152 | 72 | 1290 | 1859 | 770 | 779 | 1179 | 482 | 713 | 635 | 837 | 1106 | 1064 |
| *Diapensia lapponica* | F | 566 | 822 | 888 | 736 | 445 | 321 | 503 | 374 | 441 | 409 | *NA* | 418 | 1084 |
| *Hierochloe alpina* | I | 90 | 100 | 166 | 387 | 136 | 148 | 144 | 213 | 123 | 108 | 316 | 161 | 88 |
| *Ledum palustre* | I | 207 | 133 | 464 | 555 | 136 | 202 | 273 | 186 | 213 | 14 | 108 | 339 | 312 |
| *Luzula confusa* | I | 217 | 285 | 221 | 231 | 21 | 67 | 77 | 75 | 110 | 126 | 137 | 77 | 46 |
| *Polygonum bistorta* | I | 13 | 9 | 39 | 26 | 17 | 8 | 19 | 28 | 26 | 58 | 25 | 37 | 43 |
| *Vaccinium vitis-idaea* | I | 329 | 77 | 358 | 288 | 250 | 166 | 256 | 436 | 214 | 182 | 549 | 711 | 473 |
| **Atqasuk - Wet** |  |  |  |  |  |  |  |  |  |  |  |  |  |  |
| *Carex aquatilis* | I | 41 | 62 | 141 | 47 | 72 | 24 | 47 | 50 | 193 | 74 | 120 | *NA* | 73 |
| *Carex rariflora* | I | 32 | 20 | *NA* | 14 | *NA* | *NA* | 47 | 34 | 55 | 48 | 93 | 50 | 62 |
| *Eriophorum angustifolium* | I | 20 | 10 | 81 | 50 | 35 | 34 | 37 | *NA* | 49 | 40 | 19 | *NA* | 16 |
| *Polygonum viviparum* | I | *NA* | 14 | *NA* | *NA* | 10 | 15 | 11 | 26 | 8 | 16 | 6 | 17 | 11 |
| *Salix polaris* (female) | I | 12 | 11 | 10 | 164 | 8 | 75 | 93 | 157 | 100 | 47 | 173 | 111 | 134 |
| *Salix polaris* (male) | I | *NA* | *NA* | 10 | *NA* | 21 | 40 | 72 | 117 | 50 | 42 | 54 | 73 | 106 |
| **Utqiaġvik - Dry** |  |  |  |  |  |  |  |  |  |  |  |  |  |  |
| *Cassiope tetragona* | F | 4134 | 1890 | 1666 | 7648 | 905 | 4091 | 9158 | 6831 | 2148 | 19 | 7370 | 4334 | 5051 |
| *Luzula arctica* | I | 92 | 83 | 76 | 44 | 9 | 15 | 18 | 27 | 37 | 17 | 26 | 16 | 23 |
| *Luzula confusa* | I | 207 | 138 | 208 | 182 | 40 | 71 | 90 | 97 | 83 | 46 | 234 | 72 | 123 |
| *Papaver hultenii* | F | 27 | 43 | 43 | 34 | 35 | 64 | 37 | 45 | 20 | 14 | 21 | *6* | *NA* |
| *Potentilla hyparctica* | F | 390 | 373 | 268 | 477 | 360 | 263 | 249 | 322 | 61 | 125 | 387 | 212 | 127 |
| *Salix rotundifolia* (female) | I | 1731 | 3034 | 1858 | 2406 | 748 | 1622 | 876 | 3215 | 2282 | 1111 | 2473 | 1354 | 1272 |
| *Salix rotundifolia* (male) | I | 983 | 1342 | 1950 | 1749 | 688 | 1145 | 629 | 2338 | 1516 | 540 | 2851 | 1175 | 1185 |
| *Saxifraga punctata* | I | 133 | 107 | *NA* | *NA* | *NA* | 133 | 137 | 132 | 125 | 196 | 249 | 166 | 182 |
| *Stellaria laeta* | F | 226 | 39 | 444 | 304 | 171 | 124 | 215 | 27 | 9 | 295 | 224 | 196 | 21 |
| **Utqiaġvik - Wet** |  |  |  |  |  |  |  |  |  |  |  |  |  |  |
| *Carex aquatilis* var. *stans* | I | 258 | 87 | 49 | 97 | 110 | 123 | 97 | 200 | 113 | 37 | 650 | 63 | 106 |
| *Dupontia fisheri* | I | 166 | 10 | 434 | 234 | 288 | 414 | 285 | 28 | *NA* | 277 | 312 | 311 | 10 |
| *E. angustifolium* ssp. *triste* | I | 171 | 359 | 45 | 22 | 6 | 13 | 11 | 7 | 12 | 4 | 9 | *NA* | *NA* |
| *Hierochloe pauciflora* | I | 379 | 90 | 201 | 194 | 59 | 82 | 83 | 380 | 91 | 16 | 470 | 81 | 113 |
| *Luzula arctica* | I | 19 | 26 | 15 | 19 | 29 | 72 | 30 | 68 | 69 | 18 | 82 | 48 | 68 |
| *Luzula confusa* | I | 20 | 22 | 11 | 10 | 21 | 32 | 19 | 32 | 28 | 10 | 35 | 21 | 27 |
| *Saxifraga hieracifolia* | I | 21 | 23 | 23 | 29 | 43 | 55 | 23 | 33 | 30 | 34 | 27 | 35 | 20 |
| **M** is the unit of measurement (F – flower count; I – inflorescence count)  *NA* – not available (either less than 10 flowers occurred that year or removed due to sampling inconsistency) | | | | | | | | | | | | | | |

**SUPPLEMENTAL Figures:**


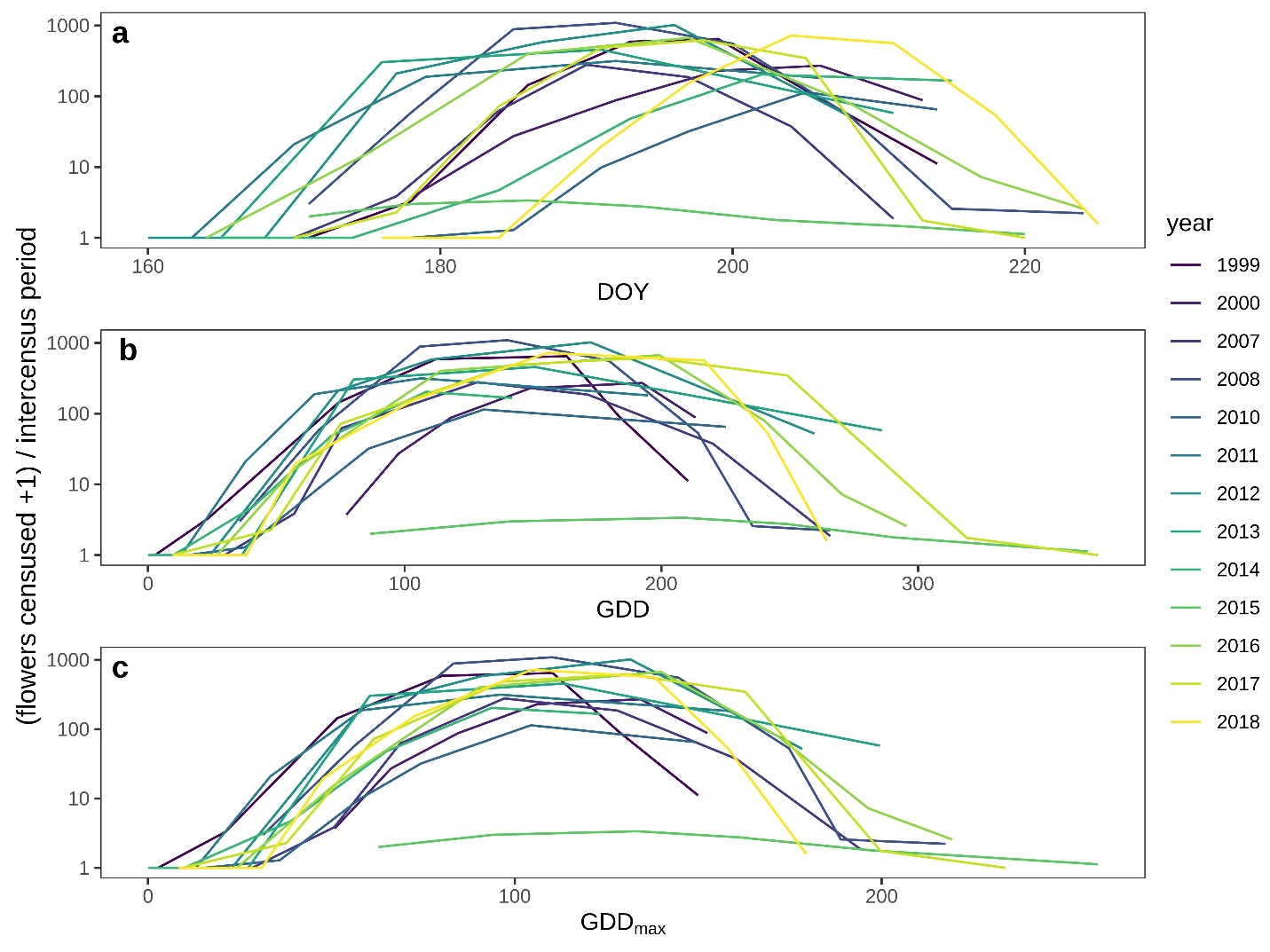


**Supplemental Fig. 1.** **Flower censuses** (log scale) plotted against the day of year (DOY; **a**), accumulated growing degree days (GDD; **b**), or accumulated growing degree days with an estimated maximum temperature threshold of 5.1^o^C (GDD_max_; **c**) for *Cassiope tetragona* at the Utqiaġvik Dry location. The greater overlap of the seasonal flower counts in **b** than **a** indicates that flowering responds to seasonal temperature more than daylength; the greater overlap of seasonal flowering counts in **c** than **b** suggests that hours warmer than 5.1^o^C do not further advance flowering phenology for this species at this location.

**Supplemental Fig. 2.** Comparison of generalized additive mixed models fitting flowering numbers versus GDD_max_ using different maximum thresholds. Each panel shows AIC values from the resulting models using maximum thresholds from 0 to 20 ^o^C (in 0.2 ^o^C increments) for a given species at a location (**a-d**); male and female flowers are presented as separate species. Red vertical lines show the estimated maximum threshold indicated by the lowest AIC values. Vertical lines are dashed if there was not consistent evidence for a meaningful maximum threshold, vertical lines are solid if, when running the model after omitting a single year, the maximum threshold was within 95% of the observed hourly summer air temperatures at the location (error bars in Fig 3).


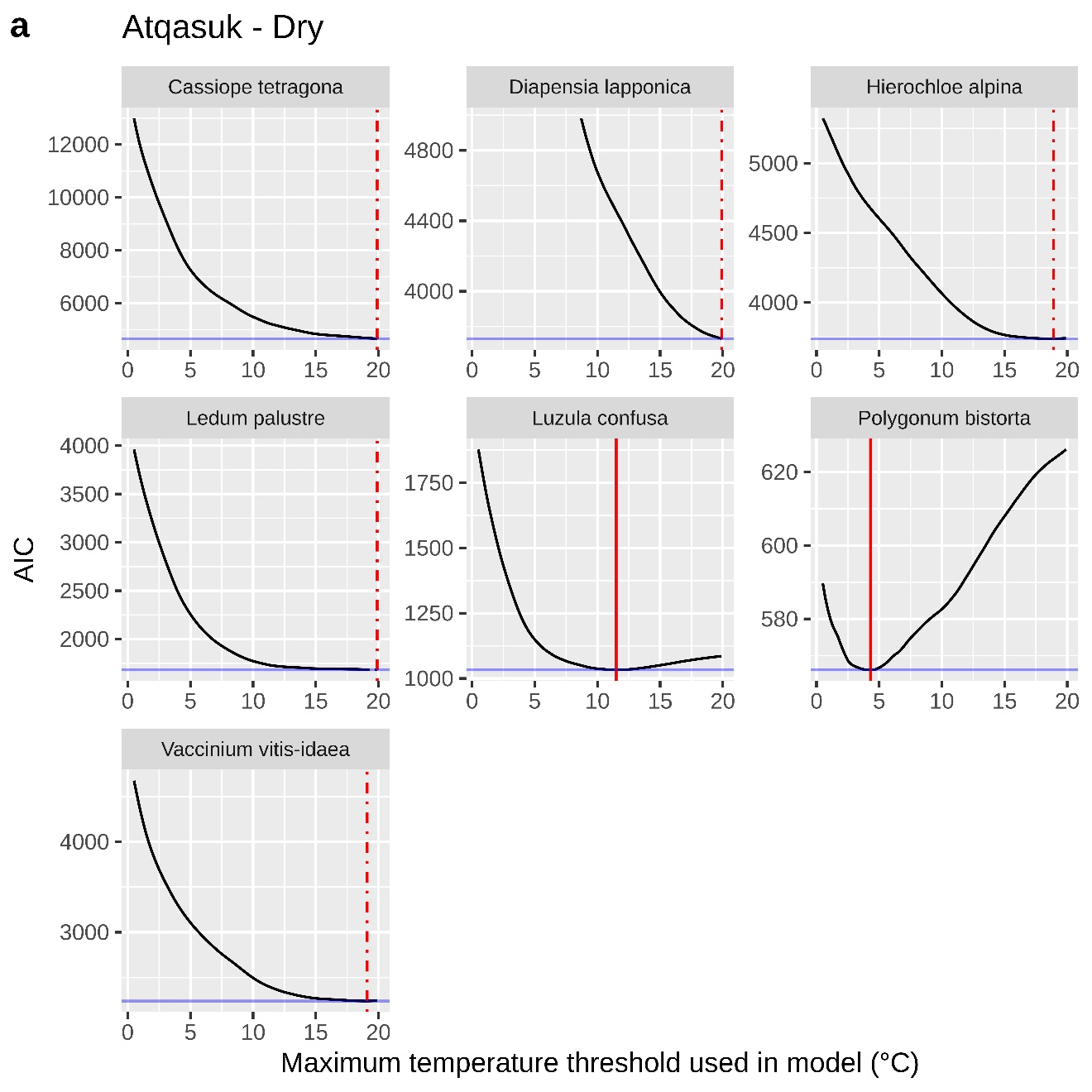

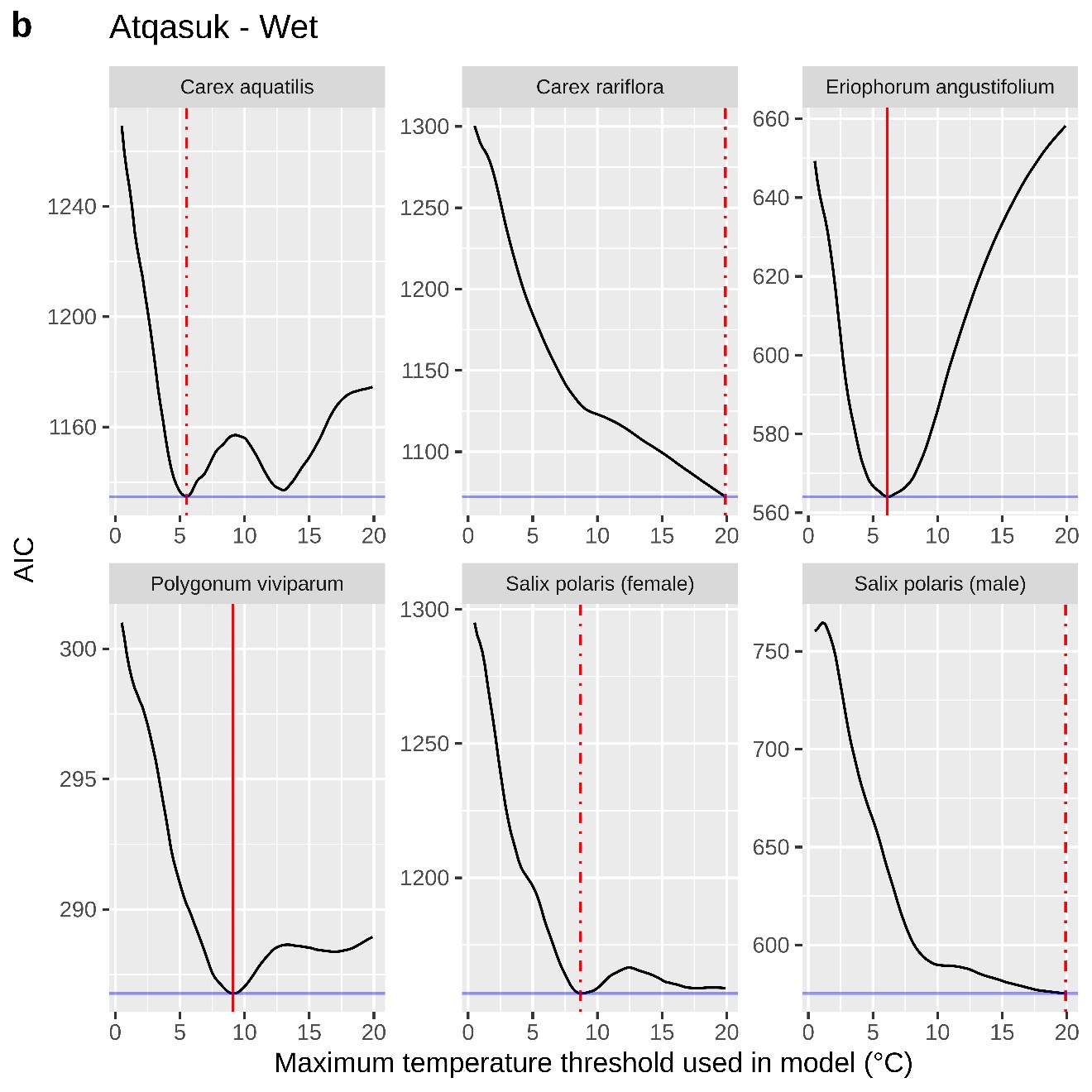

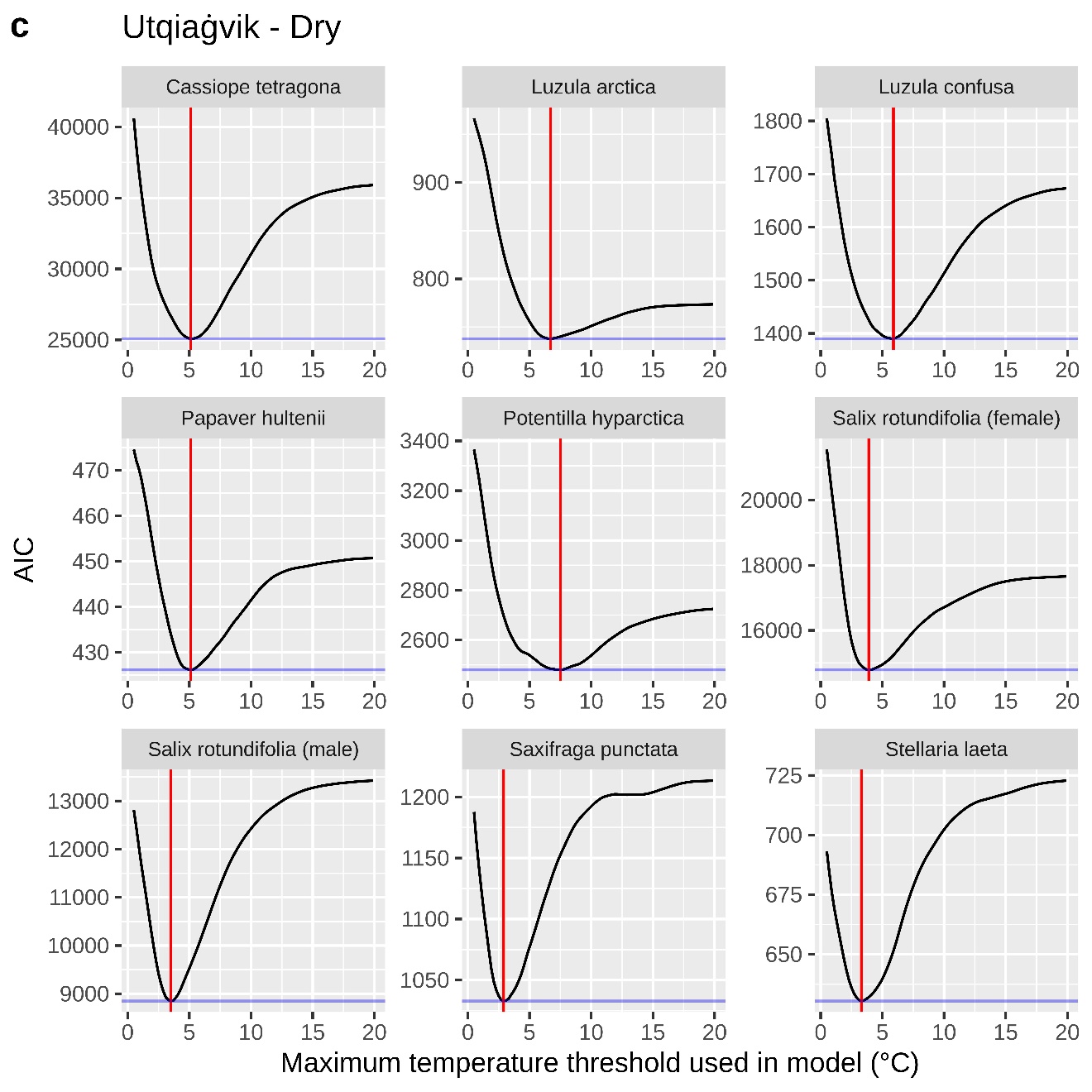

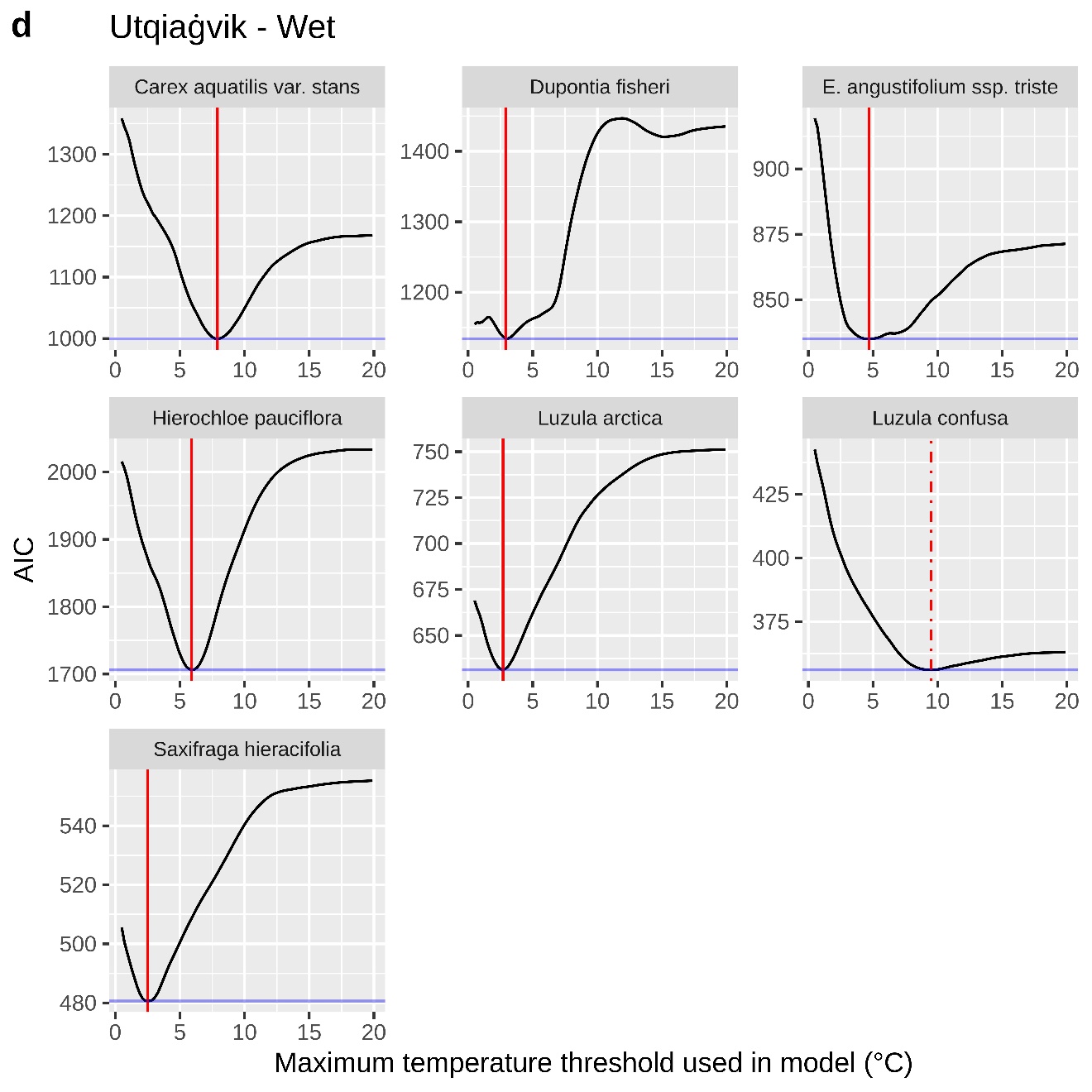

Supplement: Supplementary file 1 — Supplementary Information. [file 41598_2022_26955_MOESM1_ESM.docx]
